# Resolution of left atrial appendage thrombus with apixaban

**DOI:** 10.1186/1477-9560-11-26

**Published:** 2013-12-20

**Authors:** Tohru Kawakami, Hiroko Kobayakawa, Hiroyoshi Ohno, Nobukiyo Tanaka, Hiroki Ishihara

**Affiliations:** 1 From the Division of Cardiology, Ichinomiyanishi Hospital, 1 Kaimei-hira, Ichinomiya, Aichi, Japan

**Keywords:** Atrial fibrillation, Left atrial appendage thrombus, Apixaban

## Abstract

Left atrial appendage (LAA) thrombosis is an important cause of cardiogenic cerebral thromboembolism. Apixaban is a member of the class of novel oral anticoagulants (NOAC) and is superior to warfarin in preventing stroke or systemic embolism, causes less bleeding, and results in lower mortality in patients with atrial fibrillation. There are few reports of resolution of LAA thrombus with other NOAC. We present a 72-year-old male patient with persistent atrial fibrillation associated with left atrial thrombus. Sixteen days of apixaban treatment showed complete thrombus resolution. In this study, soluble fibrin and D-dimer levels decreased without prolongation of international normalized ratio (INR) and activated partial thromboplastin time (APTT).

## Background

Left atrial appendage (LAA) thrombus is commonly associated with atrial fibrillation (AF) and causes thromboembolic complications. Warfarin is used for prevention of thromboembolic complications with AF patients and resolution of LAA thrombus [1]. Apixaban is a member of the class of novel oral anticoagulants (NOAC). It is superior to warfarin in preventing stroke or systemic embolism, and causes less bleeding [2]. Few reports exist detailing resolution of LAA thrombus with other novel oral anticoagulants (NOAC) [3,4].

## Case presentation

**Figure 1:**
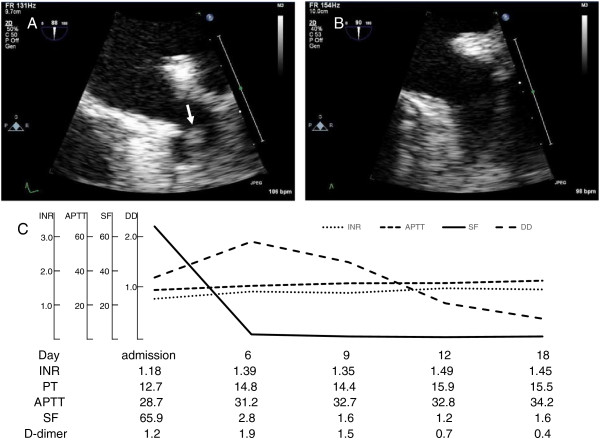
**Transesophageal echocardiography and laboratory data for the coagulation system. A)** Small thrombus formation (arrow) in the apex of the left atrial appendage (LAA) on day 4. **B)** Thrombus resolution after 16 days of anticoagulant therapy with apixaban. **C)** Plot of laboratory data for the coagulation system demonstrates a fall in SF, a rise and subsequent fall in D-dimer (DD), without prolongation of INR or APTT.

A 72-year-old male consulted our hospital due to dyspnea and palpitation, maintained for one week or more. On hospital admission the patient presented with functional NYHA class III; he had a clinical history of non-ischemic cardiomyopathy with severely impaired left ventricular function and a moderate AF-related thromboembolic risk with an actual CHA_2_DS_2_VASc score of 3, and had not been pretreated with anticoagulant. We determined that he required rapid anticoagulation to be delivered orally. We started oral anticoagulation (OAC) therapy with the direct factor Xa (FXa) inhibitor apixaban (5 mg twice daily). The patient had high levels of soluble fibrin (SF) and D-dimer, normal levels of international normalized ratio (INR) and activated partial thromboplastin time (APTT) on hospitalization. We performed transesophageal echocardiography (TEE), which revealed formation of a small thrombus (11 × 10 mm) in the left atrial appendage (LAA) (Figure 1A). After 16 days of apixaban treatment TEE showed complete thrombus resolution (Figure 1B). During apixaban treatment SF and D-dimer changed as the day passed; SF fell, while there was a rise and subsequent fall in D-dimer, without prolongation of INR or APTT (Figure 1C).

Finally, catheter ablation of persistent AF was performed successfully without clinical signs of cardiac embolism after 22 days of apixaban treatment and the patient was discharged in good medical condition under continued anticoagulant treatment with apixaban.

In this study we collected blood samples before breakfast. INR, PT, APTT, SF, and D-dimer were detectable in this trough period. All assays were performed in the laboratory of our institute. For quantitative determination of coagulation systems an automated coagulation analyzer was used (Coapresta 2000: Sekisui Medical Co., Ltd., Tokyo, Japan). PT was measured with Coagpia PT-N, whose international sensitivity index is almost equal to 1.0 (Sekisui Medical Co., Ltd.). APTT was measured with Coagpia APTT-N (Sekisui Medical Co., Ltd.). SF in sodium citrate plasma was measured with Nanopia SF (Sekisui Medical Co., Ltd.). D-dimer in sodium citrate plasma was measured with Nanopia D-dimer (Sekisui Medical Co., Ltd.). Processing and analyses were performed within 90 minutes of sample collection.

## Discussion

Apixaban is superior to warfarin in preventing stroke or systemic embolism, caused less bleeding, and resulted in lower mortality in patients with atrial fibrillation [2]. Resolution of LAA thrombus with warfarin has been previously reported [5]. There have been a few cases outlining resolution of LAA thrombus with other NOAC [3,4].

To our knowledge this is the first documented case of LAA thrombus resolution under apixaban therapy and demonstrates the changes in laboratory data for the coagulation system. The optimal therapeutic range for anticoagulation with warfarin in the prevention of thromboembolic events with non-valvular AF patients extends over INR values of 2.0 to 3.0 [6,7]. It has been reported that major bleeding is observed only at INR values above 2.5 on anticoagulation with warfarin [1]. In this study, SF and D-dimer levels decreased without prolongation of INR and APTT.

Recently, SF and D-dimer levels have been considered useful for diagnosis of thrombosis [8-14]. The TEE-guided approach with short-term anticoagulation is considered to be as safe and clinically effective as the conventional approach, and is advocated in patients in whom earlier cardioversion would be clinically beneficial [8,15,16]. Addition of the TEE-guided approach to monitoring of fibrin-related markers such as SF and D-dimer may be safer than the simple TEE-guided approach for rhythm control strategy in AF patients.

Apixaban treatment enabled thrombus resolution within a manageable time period. Therefore, this NOAC could be considered to have an important role in rhythm control strategies in similar cases. It is of undoubted interest and requires further investigation in a larger population.

In this case, we selected apixiaban. This was preferred to dabigatran treatment, which is associated with dyspepsia. Furthermore, rivaroxaban treatment was not powered for efficacy in the J-ROKET-AF study [17].

## Conclusions

We report LAA thrombus resolution with apixaban. In this study, SF and D-dimer levels decreased along with LAA thrombus resolution without prolongation of INR and APTT.

## Consent

Written informed consent was obtained from the patient for publication of this case report and any accompanying images. A copy of the written consent is available for review by the Editor-in-Chief of this journal.

## Abbreviations

LAA: Left atrial appendage; NOAC: Novel oral anticoagulants; SF: Soluble fibrin; INR: International normalized ratio; APTT: Activated partial thromboplastin time; AF: Atrial fibrillation; OAC: Oral anticoagulation; FXa: Factor Xa; TEE: Transesophageal echocardiography.

## Competing interests

Non-financial competing interests.

## Authors’ contributions

TK, MD, (Concept/design, Data analysis/interpretation). HK, MD, PhD, (Data collection). HO, MD, PhD, (Data collection). NT, MD, PhD, (Data collection). HI, MD, (Data collection). All authors read and approved the final manuscript.
